# Advanced Machine Learning to Predict Coronary Artery Disease Severity in Patients with Premature Myocardial Infarction

**DOI:** 10.31083/RCM26102

**Published:** 2025-01-16

**Authors:** Yu-Hang Wang, Chang-Ping Li, Jing-Xian Wang, Zhuang Cui, Yu Zhou, An-Ran Jing, Miao-Miao Liang, Yin Liu, Jing Gao

**Affiliations:** ^1^Thoracic Clinical College, Tianjin Medical University, 300070 Tianjin, China; ^2^School of Public Health, Tianjin Medical University, 300070 Tianjin, China; ^3^Chest Hospital, Tianjin University, 300072 Tianjin, China; ^4^Department of Cardiology, Tianjin Chest Hospital, 300222 Tianjin, China; ^5^Cardiovascular Institute, Tianjin Chest Hospital, 300222 Tianjin, China; ^6^Tianjin Key Laboratory of Cardiovascular Emergency and Critical Care, 300070 Tianjin, China

**Keywords:** premature myocardial infarction, machine learning, prediction system

## Abstract

**Background:**

Studies using machine learning to identify the target characteristics and develop predictive models for coronary artery disease severity in patients with premature myocardial infarction (PMI) are limited.

**Methods:**

In this observational study, 1111 PMI patients (≤55 years) at Tianjin Chest Hospital from 2017 to 2022 were selected and divided according to their SYNTAX scores into a low-risk group (≤22) and medium–high-risk group (>22). These groups were further randomly assigned to a training or test set in a ratio of 7:3. Lasso–logistic was initially used to screen out target factors. Subsequently, Lasso–logistic, random forest (RF), k-nearest neighbor (KNN), support vector machine (SVM), and eXtreme Gradient Boosting (XGBoost) were used to establish prediction models based on the training set. After comparing prediction performance, the best model was chosen to build a prediction system for coronary artery severity in PMI patients.

**Results:**

Glycosylated hemoglobin (HbA1c), angina, apolipoprotein B (ApoB), total bile acid (TBA), B-type natriuretic peptide (BNP), D-dimer, and fibrinogen (Fg) were associated with the severity of lesions. In the test set, the area under the curve (AUC) of Lasso–logistic, RF, KNN, SVM, and XGBoost were 0.792, 0.775, 0.739, 0.656, and 0.800, respectively. XGBoost showed the best prediction performance according to the AUC, accuracy, F1 score, and Brier score. In addition, we used decision curve analysis (DCA) to assess the clinical validity of the XGBoost prediction model. Finally, an online calculator based on the XGBoost was established to measure the severity of coronary artery lesions in PMI patients

**Conclusions:**

In summary, we established a novel and convenient prediction system for the severity of lesions in PMI patients. This system can swiftly identify PMI patients who also have severe coronary artery lesions before the coronary intervention, thus offering valuable guidance for clinical decision-making.

## 1. Introduction

Acute myocardial infarction (AMI) has experienced a shift in its incidence trend among different populations, primarily attributed to modern economic development, lifestyle modifications, and environmental climate changes [1, 2, 3]. Notably, in the United States, there has been an increasing proportion of young patients affected by AMI [4]. Similarly, the AMI incidence rate in China has yet to reduce, with the most significant rise observed among younger populations, particularly in males [5]. Consequently, there has been a growing interest among researchers in investigating premature acute myocardial infarction (PMI) in young individuals; most studies now define the age of onset for PMI as ranging between 45 and 55 years [2, 6].

Recent reports have highlighted significant differences in the clinical course, risk factors, and characteristics of coronary artery lesions between PMI and myocardial infarction in the older population. Specifically, a majority of PMI patients exhibit comorbidities, along with higher rates of multibranch lesions and an increased occurrence of in-hospital and out-of-hospital major adverse cardiovascular events (MACEs) [7, 8, 9]. Patients with PMI exhibit a higher likelihood of obesity, a history of smoking, previous hypertension, and abnormalities in glucose and lipid metabolism compared to older patients [10, 11, 12, 13, 14].

Based on the anatomical characteristics of coronary artery lesions, the SYNTAX score is a scoring system for risk stratification in patients with coronary artery disease (CAD). When the SYNTAX score is high, it often represents a multibranch or occlusive lesion in the coronary artery or even a poor prognosis, meaning it is an effective tool for customized revascularization therapy for individual patients [15]. This study used the SYNTAX score to quantitatively assess the extent of coronary artery lesions in patients with PMI [16].

There is a lack of studies with larger sample sizes that utilize multiple machine learning approaches to predict the severity of coronary lesions in patients with PMI. Therefore, this study aimed to assess the influential factors of coronary lesion severity and their respective contributions in PMI patients. Additionally, we intended to develop a machine learning-based risk prediction system for lesion severity in PMI. The primary goal was to efficiently identify high-risk groups with more severe lesions in PMI patients before coronary intervention. By achieving this, there is the potential to provide valuable decision-making guidance for precise diagnosis and treatment, ultimately improving in-hospital and long-term outcomes for PMI patients.

## 2. Materials and Methods

### 2.1 Study Population

**Fig. 1.**
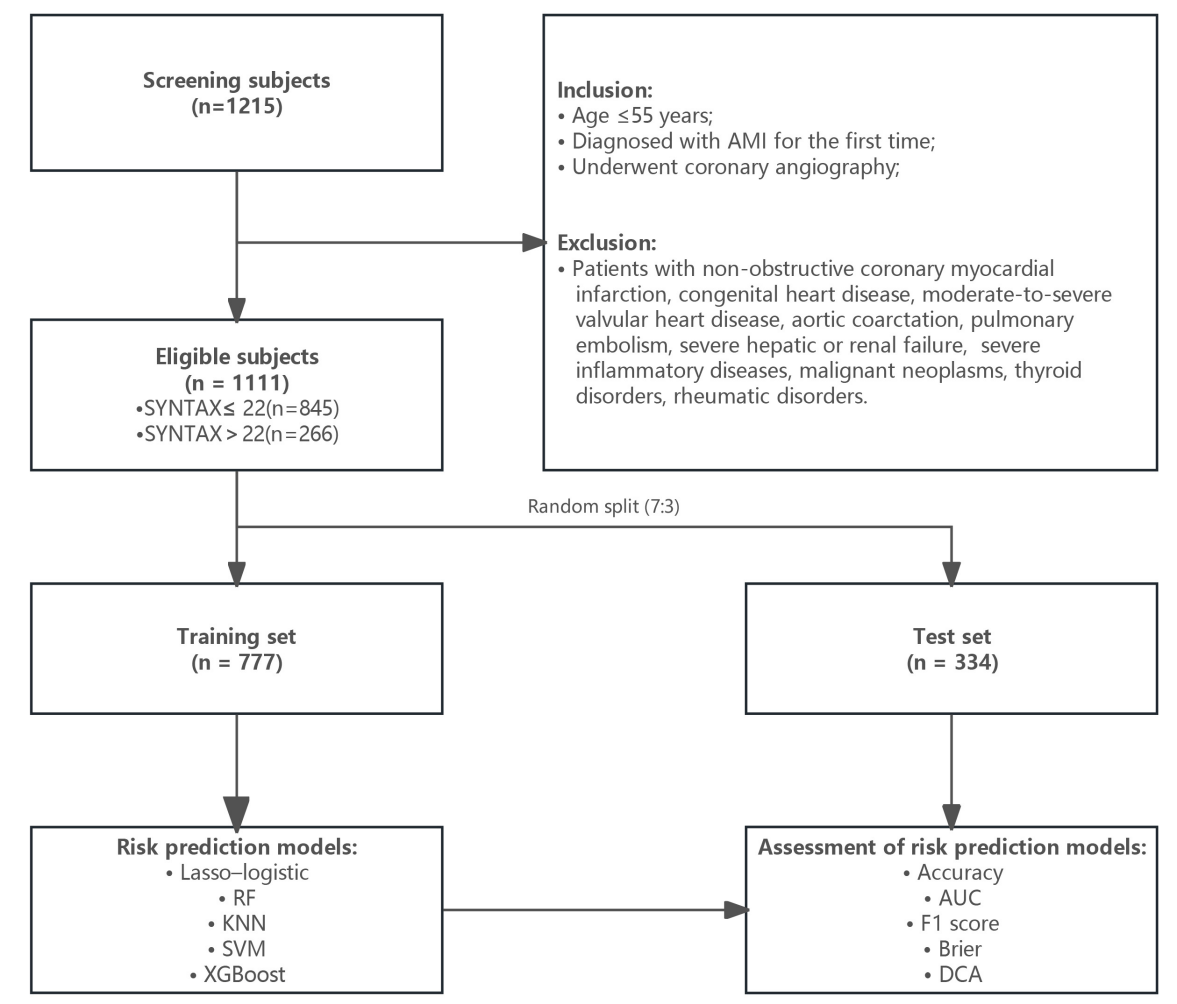
**A flowchart describing the general framework of the study**. RF, random forest; KNN, k-nearest neighbor; SVM, support vector machine; AUC, area under the curve; DCA, decision curve analysis; AMI, acute myocardial infarction.

In this observational study, 1111 patients with PMI (≤55 years old) who underwent coronary angiography at Tianjin Chest Hospital from January 2017 to December 2022 were consecutively enrolled to establish a PMI database. Subsequently, they were randomly divided into a training set (n = 777) and a test set (n = 334) in a ratio of 7:3. The study process is illustrated in Fig. 1. The severity of coronary lesions based on the SYNTAX score was utilized to classify PMI patients into a low-risk group (SYNTAX ≤22) and a medium–high-risk group (SYNTAX >22) [17].

PMI comprises early-onset non-ST-segment elevation myocardial infarction (NSTEMI) and acute ST-segment elevation myocardial infarction (STEMI). The diagnostic criteria for PMI require the presence of acute myocardial injury with clinical evidence of acute myocardial ischemia, which can be indicated by the detection of troponin elevation and/or reduction (exceeding the upper 99th percentile of the reference value on at least one occasion), as well as one of the following: evidence of myocardial ischemia, new-onset ischemic electrocardiographic changes, new pathologic Q waves, imaging suggestive of deletion or segmental ventricular wall motion abnormalities in the latest onset of surviving myocardium consistent with ischemia, and coronary thrombosis confirmed by coronary angiography [18, 19, 20].

Exclusion criteria were the presence of any of the following: non-obstructive coronary myocardial infarction, congenital heart disease, moderate-to-severe valvular heart disease, aortic coarctation, pulmonary embolism, patients with severe hepatic or renal failure, severe inflammatory diseases, malignant neoplasms, thyroid disorders, rheumatologic diseases, and patients who did not undergo coronary angiography.

The study protocol was approved by the Internal Review Board of Tianjin Chest Hospital (No. 2017 KY-007-01), and all included patients provided signed informed consent before participating in the study. All procedures were in accordance with the ethical standards of the Declaration of Helsinki and its subsequent amendments or similar ethical standards.

### 2.2 Data Collection

We extracted clinical information on the included PMI patients from the electronic medical record system, including their demographic characteristics, past medical history, and laboratory data. These data were then collated into the pre-designed case report form (CRF) in accordance with the study protocol. We utilized Epidata (Version 3.1; The Epidata Association, Odense, Denmark) for data entry, quality control, and consistency testing to ensure standardized data management, ultimately establishing the PMI database. A total of 63 variables were screened from this database for this study, including gender, age, smoking, alcohol consumption, past medical history (hypertension, diabetes, hyperlipidemia, cerebrovascular disease, angina), family history of CAD, Killip classification, type of myocardial infarction (MI), laboratory investigations (blood counts, C-reactive protein, neutrophil-to-lymphocyte ratio (NLR), platelet-to-lymphocyte ratio (PLR), monocyte-to-lymphocyte ratio (MLR), C-reactive protein-to-lymphocyte ratio (CLR), systemic inflammation response index (SIRI), systemic immunoinflammatory index (SII), HbA1c (glycosylated hemoglobin), lipids, RC (residual cholesterol), non-HDL (non-high-density lipoprotein), TyG (triglyceride–glucose index), HCY (hepatic and renal function, homocysteine), cardiac enzymes, BNP (B-type natriuretic peptide), and coagulation function). The data absence rate was less than 5% for each variable; we used the median to fill in the missing values for continuous variables and conducted multiple imputations for categorical variables.

TyG = LN (triglycerides (mg/dL) × glucose (mg/dL)/2); RC = total cholesterol – HDL cholesterol – LDL cholesterol; non-HDL cholesterol = total cholesterol – HDL cholesterol; SIRI = monocyte × neutrophil/lymphocyte; SII = platelet × neutrophil/lymphocyte.

### 2.3 SYNTAX Score

Coronary angiography (CAG) was performed by two experienced interventional cardiologists who conducted the procedure and analyzed the angiographic images. The SYNTAX scoring system utilizes a 16-segment approach to assess the coronary tree, considering the dominant type of coronary artery, lesion location, degree of stenosis, and lesion characteristics for evaluating lesions with a diameter ≥1.5 mm and ≥50% stenosis in the coronary artery. The scoring algorithm comprises 12 items, with the first three relating to the dominant type of coronary artery, number of lesions, and number of diseased vessel segments, while the remaining nine pertain to lesion characteristics (complete occlusion, truncus, bifurcation, aorta, open lesion, severe tortuosity, lesion length ≥20 mm, severe calcification, thrombus, and diffuse small-vessel lesion). The SYNTAX score is obtained by summing the individual scores for each lesion. In cases where multiple lesions are present within a segment, one lesion is scored if the distance is less than three times the reference diameter, and two lesions are scored if the distance exceeds three times the reference diameter [21].

### 2.4 Statistical Analysis

We performed statistical analysis using SPSS statistical software (Version 26.0; IBM, Armonk, NY, USA) and R statistical software (Version 4.3.1; PBC, Boston, MA, USA). Categorical variables were presented as n (%), and differences between groups were assessed using the chi-square or Fisher’s exact test. Continuous variables with a normal distribution and homogeneous variance were presented as the mean ± standard deviation, and differences between groups were evaluated using the independent samples *t*-test. For continuous variables that did not follow a normal distribution or have homogeneous variance, data were presented as the median (interquartile range), and the nonparametric rank sum test was used to assess the significance of differences between groups. Statistical significance was defined as *p* < 0.05.

This study initially employed Lasso–logistic regression to screen variables for inclusion in the machine learning model. Subsequently, five machine learning methods, namely Lasso–logistic, random forest (RF), k-nearest neighbor (KNN), support vector machine (SVM), and XGBoost, were utilized to develop predictive models based on the training set. To select the optimal hyperparameters to improve the performance of the model, we employed a random search for hyperparameter optimization in machine learning. We used K-fold cross-validation (K = 5) to perform internal validation in the training set and an independent test set to evaluate the performance of the machine learning model. The predictive performance was assessed using receiver operating characteristic (ROC) curves, and the area under the curve (AUC) of the ROC, accuracy, F1 scores, and Brier scores of different predictive models were analyzed using the test set to identify the most effective model. Additionally, decision curve analysis (DCA) was employed to evaluate the clinical validity of the selected predictive models. Ultimately, a risk prediction system for lesion severity in patients with PMI was established.

Among them, the estimation of sample content belongs to the multifactorial prediction model [22]. Based on the overall study design, considering the shrinkage factor, prevalence rate, and C-statistic-related indices, we utilized the pmsampsize package in R software to calculate the sample size. The results indicated that our sample size sufficiently met the requirements for establishing the prediction model with the current variables.

## 3. Results

A total of 1111 patients with PMI who underwent coronary angiography were included in this study. Based on the SYNTAX score, participants were divided into a low-risk group (SYNTAX ≤22, n = 845) and a medium–high-risk group (SYNTAX >22, n = 266). To facilitate the rapid application of the prediction system to the clinical setting, we simplified the interpretation and application of the model in the clinical setting by converting continuous variables to categorical variables and using the median as the cutoff value (Table 1).

**Table 1.** **Baseline characteristics of all PMI patients according to SYNTAX score**.

Variables			Overall	SYNTAX ≤22	SYNTAX >22	*p*-value
			(n = 1111)	(n = 845)	(n = 266)	
	Age, years		42.0 (38.0, 44.0)	42.0 (37.5, 44.0)	42.0 (38.0, 45.0)	0.446
	Age >43 years, (%)		490 (44.1)	364 (43.1)	126 (47.4)	0.247
	Male, (%)		1013 (91.2)	775 (91.7)	238 (89.5)	0.317
	Smoking, (%)		785 (70.7)	600 (71.0)	185 (69.5)	0.705
	Drinking, (%)		409 (36.8)	327 (38.7)	82 (30.8)	0.025*
Past history, (%)						
	Hypertension		533 (48.0)	406 (48.0)	127 (47.7)	0.987
	Diabetes		235 (21.2)	163 (19.3)	72 (27.1)	0.009*
	Hyperlipidemia		276 (24.8)	203 (24.0)	73 (27.4)	0.296
	Stroke		38 (3.4)	26 (3.1)	12 (4.5)	0.353
	Angina		178 (16.0)	116 (13.7)	62 (23.3)	<0.001**
	Family history of CAD		117 (10.5)	91 (10.8)	26 (9.8)	0.729
Killip classification, (%)						0.227
	I		1072 (96.5)	819 (96.9)	253 (95.1)	
	≥II		39 (3.5)	26 (3.1)	13 (4.9)	
Type of MI, (%)						0.882
	STEMI		862 (77.6)	657 (77.8)	205 (77.1)	
	NSTEMI		249 (22.4)	188 (22.2)	61 (22.9)	
Laboratory data						
	Blood routine					
		WBC >10.23 × 10^9^/L, (%)	555 (50.0)	421 (49.8)	134 (50.4)	0.931
		Neutrophil % >73.43%, (%)	555 (50.0)	418 (49.5)	137 (51.5)	0.611
		Lymphocyte % >18.40%, (%)	554 (49.9)	431 (51.0)	123 (46.2)	0.199
		Monocyte % >6.00%, (%)	551 (49.6)	407 (48.2)	144 (54.1)	0.104
		Neutrophil >7.50 × 10^9^/L, (%)	554 (49.9)	419 (49.6)	135 (50.8)	0.794
		Lymphocyte >1.83 × 10^9^/L, (%)	556 (50.0)	430 (50.9)	126 (47.4)	0.352
		Monocyte >0.60 × 10^9^/L, (%)	516 (46.4)	380 (45.0)	136 (51.1)	0.092
		RBC >4.88 × 10^12^/L, (%)	549 (49.4)	426 (50.4)	123 (46.2)	0.264
		Hb >148.00 g/L, (%)	533 (48.0)	418 (49.5)	115 (43.2)	0.088
		PLT >240.00 × 10^9^/L, (%)	554 (49.9)	417 (49.3)	137 (51.5)	0.587
	Inflammation indicators					
		CRP >5.47 mg/L, (%)	553 (49.8)	398 (47.1)	155 (58.3)	0.002*
		NLR >4.00, (%)	554 (49.9)	412 (48.8)	142 (53.4)	0.213
		PLR >131.49, (%)	556 (50.0)	422 (49.9)	134 (50.4)	0.957
		MLR >0.33, (%)	555 (50.0)	409 (48.4)	146 (54.9)	0.076
		CLR >3.11, (%)	555 (50.0)	407 (48.2)	148 (55.6)	0.040*
		SIRI >2.48, (%)	556 (50.0)	416 (49.2)	140 (52.6)	0.370
		SII >960.73, (%)	556 (50.0)	418 (49.5)	138 (51.9)	0.538
	Glycolipid metabolism indicators					
		HbA1c >5.80%, (%)	466 (41.9)	270 (32.0)	196 (73.7)	<0.001**
		Glu >5.77 mmol/L, (%)	556 (50.0)	407 (48.2)	149 (56.0)	0.031*
		TC >4.81 mmol/L, (%)	552 (49.7)	413 (48.9)	139 (52.3)	0.373
		TG >2.02 mmol/L, (%)	548 (49.3)	420 (49.7)	128 (48.1)	0.704
		HDL >0.92 mmol/L, (%)	536 (48.2)	400 (47.3)	136 (51.1)	0.313
		LDL >3.19 mmol/L, (%)	555 (50.0)	408 (48.3)	147 (55.3)	0.055
		VLDL >0.56 mmol/L, (%)	535 (48.2)	410 (48.5)	125 (47.0)	0.715
		non-HDL >3.86 mmol/L, (%)	554 (49.9)	412 (48.8)	142 (53.4)	0.213
		RC >0.56 mmol/L, (%)	542 (48.8)	416 (49.2)	126 (47.4)	0.646
		TC/HDL >5.21, (%)	556 (50.0)	427 (50.5)	129 (48.5)	0.611
		TG/HDL >2.21, (%)	556 (50.0)	417 (49.3)	139 (52.3)	0.449
		LDL/HDL >3.46, (%)	557 (50.1)	417 (49.3)	140 (52.6)	0.388
		ApoA1 >1.10 g/L, (%)	512 (46.1)	390 (46.2)	122 (45.9)	0.990
		ApoB >1.13 g/L, (%)	545 (49.1)	386 (45.7)	159 (59.8)	<0.001**
		ApoA1/ApoB >0.98, (%)	580 (52.2)	460 (54.4)	120 (45.1)	0.010*
		TyG >9.18, (%)	555 (50.0)	414 (49.0)	141 (53.0)	0.284
	Kidney function indicators					
		Urea >4.30 mmol/L, (%)	542 (48.8)	402 (47.6)	140 (52.6)	0.171
		Cr >75.00 µmol/L, (%)	554 (49.9)	411 (48.6)	143 (53.8)	0.166
		UA >360.00 µmol/L, (%)	554 (49.9)	432 (51.1)	122 (45.9)	0.154
	Liver function indicators					
		TBA >1.47 µmol/L, (%)	553 (49.8)	442 (52.3)	111 (41.7)	0.003*
		TBil >13.80 µmol/L, (%)	552 (49.7)	429 (50.8)	123 (46.2)	0.223
		DBil >5.00 µmol/L, (%)	496 (44.6)	381 (45.1)	115 (43.2)	0.645
		ALT >42.80 U/L, (%)	554 (49.9)	415 (49.1)	139 (52.3)	0.410
		AST >106.55 U/L, (%)	555 (50.0)	419 (49.6)	136 (51.1)	0.713
		LDH >438.00 U/L, (%)	556 (50.0)	411 (48.6)	145 (54.5)	0.110
		α-HBDH >400.50 U/L, (%)	556 (50.0)	413 (48.9)	143 (53.8)	0.187
	HCY >12.70 µmol/L, (%)		548 (49.3)	403 (47.7)	145 (54.5)	0.062
	Cardiac function indicators					
		CK >1017 U/L, (%)	555 (50.0)	418 (49.5)	137 (51.5)	0.611
		CK-MB >88 U/L, (%)	551 (49.6)	421 (49.8)	130 (48.9)	0.841
		TnT >1.94 ug/L, (%)	554 (49.9)	416 (49.2)	138 (51.9)	0.494
		BNP >269.75 pg/mL, (%)	554 (49.9)	385 (45.6)	169 (63.5)	<0.001**
	Coagulation indicators					
		D-dimer >0.28 mg/L, (%)	534 (48.1)	370 (43.8)	164 (61.7)	<0.001**
		Fg >3.30 g/L, (%)	556 (50.0)	386 (45.7)	170 (63.9)	<0.001**
SYNTAX score			16.5 (10.0, 22.0)	13.5 (9.0, 18.0)	26.5 (24.0, 31.6)	<0.001**

Notes: *p < 0.05, **p < 0.001. PMI, premature myocardial infarction; CAD, coronary artery disease; MI, myocardial infarction; STEMI, ST-segment elevation myocardial infarction; NSTEMI, non-ST-segment elevation myocardial infarction; WBC, white blood cells; RBC, red blood cells; Hb, hemoglobin; PLT, platelet; CRP, C-reactive protein; NLR, neutrophil-to-lymphocyte ratio; PLR, platelet-to-lymphocyte ratio; MLR, monocyte-to-lymphocyte ratio; CLR, C-reactive protein-to-lymphocyte ratio; SIRI, systemic inflammation response index; SII, systemic immune-inflammation index; HbA1c, glycosylated hemoglobin; Glu, glucose; TC, total cholesterol; TG, triglycerides; HDL, high-density lipoprotein; LDL, low-density lipoprotein; VLDL, very-low-density lipoprotein; non-HDL, non-high-density lipoprotein; RC, residual cholesterol; ApoA1, apolipoprotein A1; ApoB, apolipoprotein B; TyG, triglyceride glucose index; Cr, creatinine; UA, uric acid; TBA, total bile acid; TBil, total bilirubin; DBil, direct bilirubin; ALT, alanine aminotransferase; AST, aspartate aminotransferase; LDH, lactate dehydrogenase; α-HBDH, alpha-hydroxybutyrate dehydrogenase; HCY, homocysteine; CK, creatine kinase; CK-MB, creatine kinase MB; TnT, troponin T; BNP, B-type natriuretic peptide; Fg, fibrinogen.

### 3.1 Patient Characteristics

Table 1 compares the clinical characteristics of patients in the medium–high SYNTAX score group and low SYNTAX score group. In this study, the majority of participants were male, with 1013 males (91.2%) and 98 females (8.8%). Compared to the low SYNTAX score group, the medium–high SYNTAX score group had a higher prevalence of individuals with a history of diabetes (*p* = 0.009) and angina (*p* < 0.001). Additionally, there was a significantly higher proportion of high-value levels of CLR (*p* = 0.040), HbA1c (*p* < 0.001), glucose (Glu) (*p* = 0.031), apolipoprotein B (ApoB) (*p* < 0.001), C-reactive protein (CRP) (*p* = 0.002), BNP (*p* < 0.001), D-dimer (*p* < 0.001), and fibrinogen (Fg) (*p* < 0.001) in the medium–high SYNTAX score group, while total bile acid (TBA) (*p* = 0.003) and apolipoprotein A1 (ApoA1)/ApoB (*p* = 0.010) showed the opposite trend.

**Supplementary Table 1** presents the baseline table of raw continuous variables for laboratory data in PMI patients. Our findings revealed that compared to the low group, patients in the medium–high SYNTAX group exhibited significantly elevated levels of MLR (*p* = 0.024), CLR (*p* = 0.008), HbA1c (*p* < 0.001), Glu (*p* = 0.001), ApoB (*p* = 0.003), lactate dehydrogenase (LDH) (*p* = 0.049), alpha-hydroxybutyrate dehydrogenase (α-HBDH) (*p* = 0.046), CRP (*p* = 0.016), BNP (*p* < 0.001), and Fg (*p* < 0.001), whereas the opposite was true for ApoA1/ApoB (*p* = 0.012) and TBA (*p* = 0.034). We observed significant differences in angina, HbA1c, BNP, and Fg levels in the baseline tables for both the training and test sets (**Supplementary Table 2**). No significant differences were found for any of the variables (*p* > 0.05) between the training and test sets, except for HbA1c (*p* = 0.005), HDL (*p* = 0.041), and aspartate aminotransferase (AST) (*p* = 0.023) (**Supplementary Table 3**).

### 3.2 Factors Influencing the Severity of Coronary Artery Lesions in Patients with PMI

**Fig. 2.**
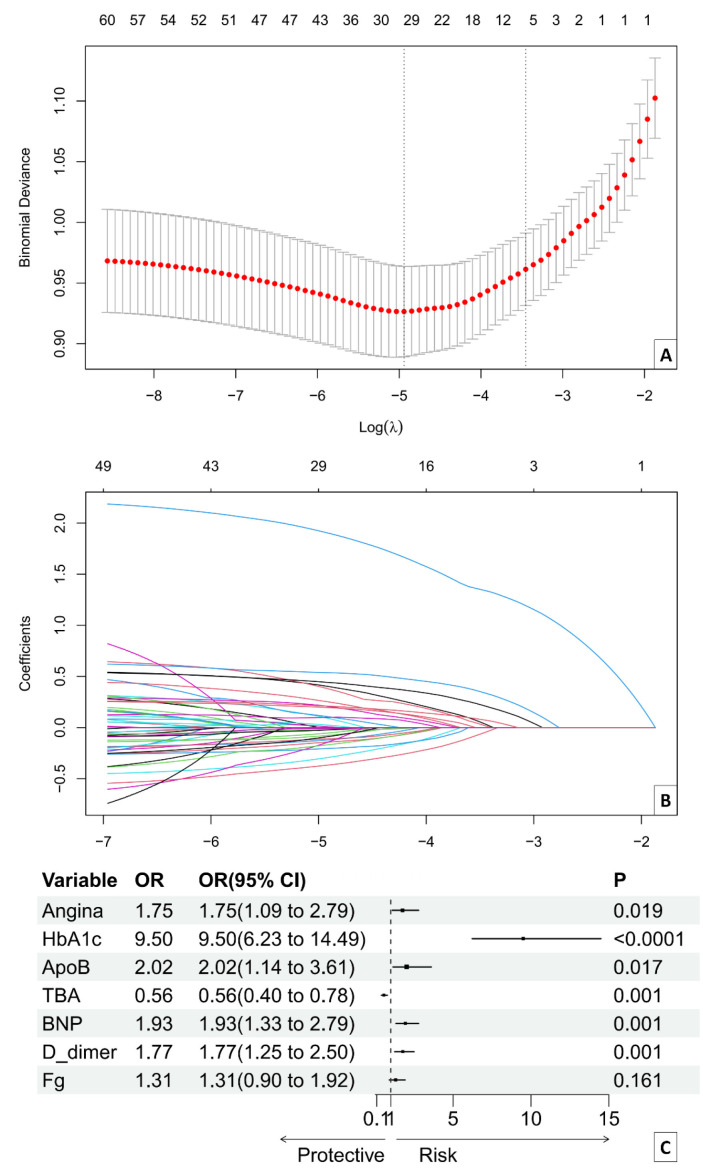
**Identification of factors influencing the severity of coronary artery lesions in patients with PMI**. (A) Identification of the optimal penalization estimate of lambda in the Lasso regression. (B) Lasso estimate profile of the predictive variables. (C) The forest plot of the logistic regression. HbA1c, glycosylated hemoglobin; ApoB, apolipoprotein B; TBA, total bile acid; BNP, B-type natriuretic peptide; Fg, fibrinogen; OR, odds ratio.

Lasso–logistic was employed to identify significant coronary artery lesion severity predictors. Seven variables were screened using Lasso–logistic, including angina, HbA1c, TBA, ApoB, BNP, D-dimer, and Fg (Fig. 2A,B). HbA1c (odds ratio, OR 9.50, 95% CI 6.23–14.49, *p* < 0.001), BNP (OR 1.93, 95% CI 1.33–2.79, *p* = 0.001), D-dimer (OR 1.77, 95% CI 1.25–2.50, *p* = 0.001), ApoB (OR 2.02, 95% CI 1.14–3.61, *p* = 0.017), TBA (OR 0.56, 95% CI 0.40–0.78, *p* = 0.001), angina (OR 1.75, 95% CI 1.09–2.79, *p* = 0.019), and Fg (OR 1.31, 95% CI 0.90–1.92, *p* = 0.161) were identified as significant predictors (Fig. 2C). Angina, BNP, HbA1c, ApoB, D-dimer, and Fg were among the risk factors for coronary artery disease. TBA was found to be a protective factor against coronary artery lesions.

### 3.3 Establishment and Evaluation of a Model for Predicting the Severity of Coronary Artery Lesions in PMI Patients

**Fig. 3.**
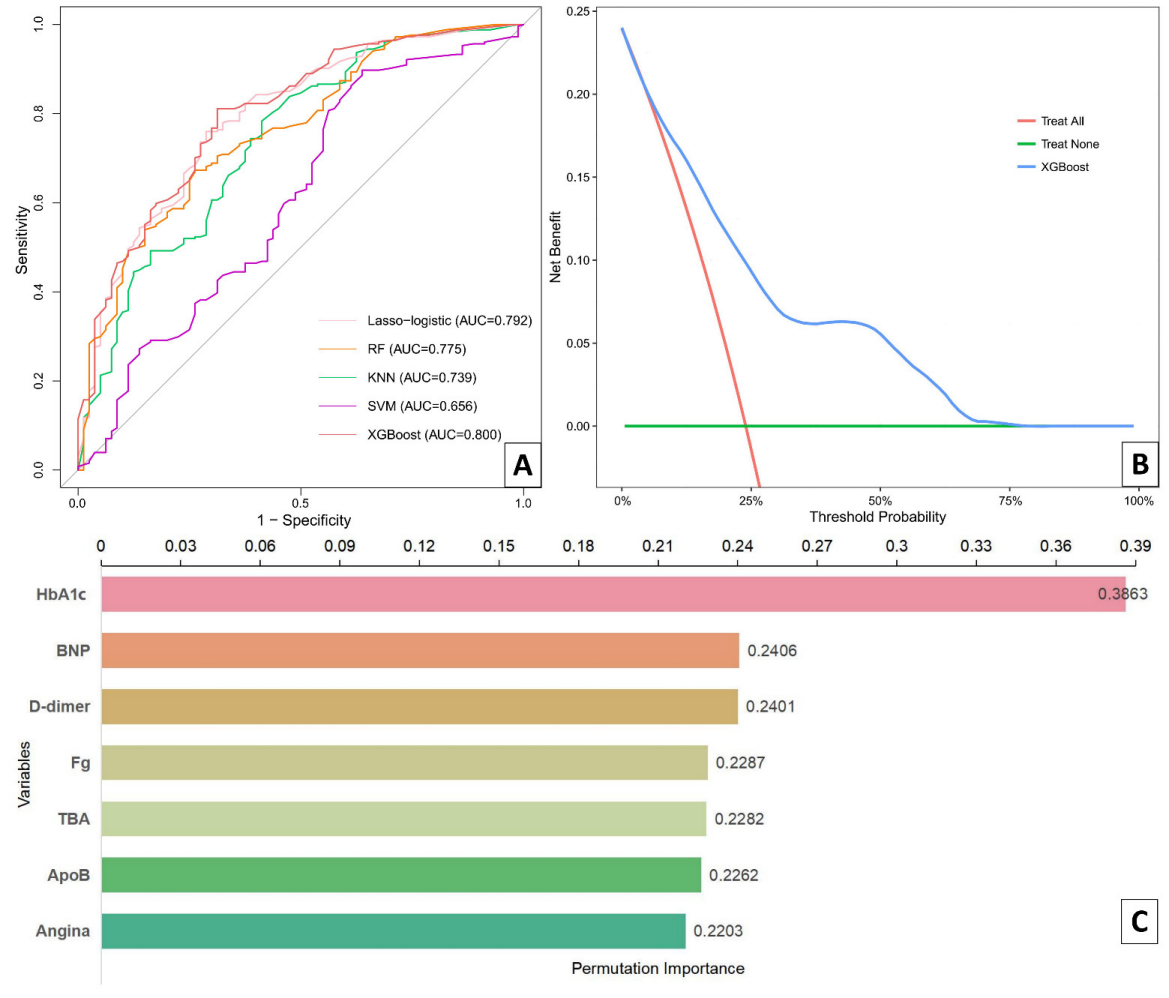
**Evaluation of predictive models and importance of variables for assessing coronary artery lesion severity in PMI patients**. (A) ROC curves from the testing set using different machine learning algorithms. (B) Decision curve analysis of the XGBoost data in the test set. (C) The relative importance of predictors in the XGBoost data. HbA1c, glycosylated hemoglobin; ApoB, apolipoprotein B; TBA, total bile acid; BNP, B-type natriuretic peptide; Fg, fibrinogen; ROC, receiver operating characteristic; RF, random forest; KNN, k-nearest neighbor; SVM, support vector machine; AUC, area under the curve; PMI, premature myocardial infarction.

Five machine learning methods, Lasso–logistic, RF, KNN, SVM, and XGBoost, were used to incorporate the seven variables selected using the initial Lasso–logistic regression model to develop a prediction model based on the training set data. The ROC curves of each model were analyzed (Fig. 3A), revealing that their respective AUCs were as follows: 0.792, 0.775, 0.739, 0.656, and 0.800, respectively. Among them, XGBoost demonstrated the highest performance, making it the most optimal prediction model (accuracy = 0.817, AUC = 0.800, F1 score = 0.771, and Brier = 0.142) (Table 2). We employed DCA to assess the clinical validity of this predictive model, and the net benefit of the test set for the predictive model was significantly higher compared to the two extreme cases (Fig. 3B). Moreover, the XGBoost model ranked the variables in terms of their importance (Fig. 3C), with HbA1c identified as the most crucial factor.

**Table 2.** **An assessment of the effectiveness of models constructed using five types of machine learning**.

	Accuracy	AUC	F1 score	Brier
Lasso–logistic	0.793	0.792	0.617	0.145
RF	0.790	0.775	0.692	0.150
KNN	0.766	0.739	0.514	0.161
SVM	0.763	0.656	0.509	0.169
XGBoost	0.817	0.800	0.771	0.142

Notes: RF, random forest; KNN, k-nearest neighbor; SVM, support vector machine; AUC, area under the curve.

### 3.4 A Prediction System for the Severity of Coronary Artery Lesions in Patients with PMI

**Fig. 4.**
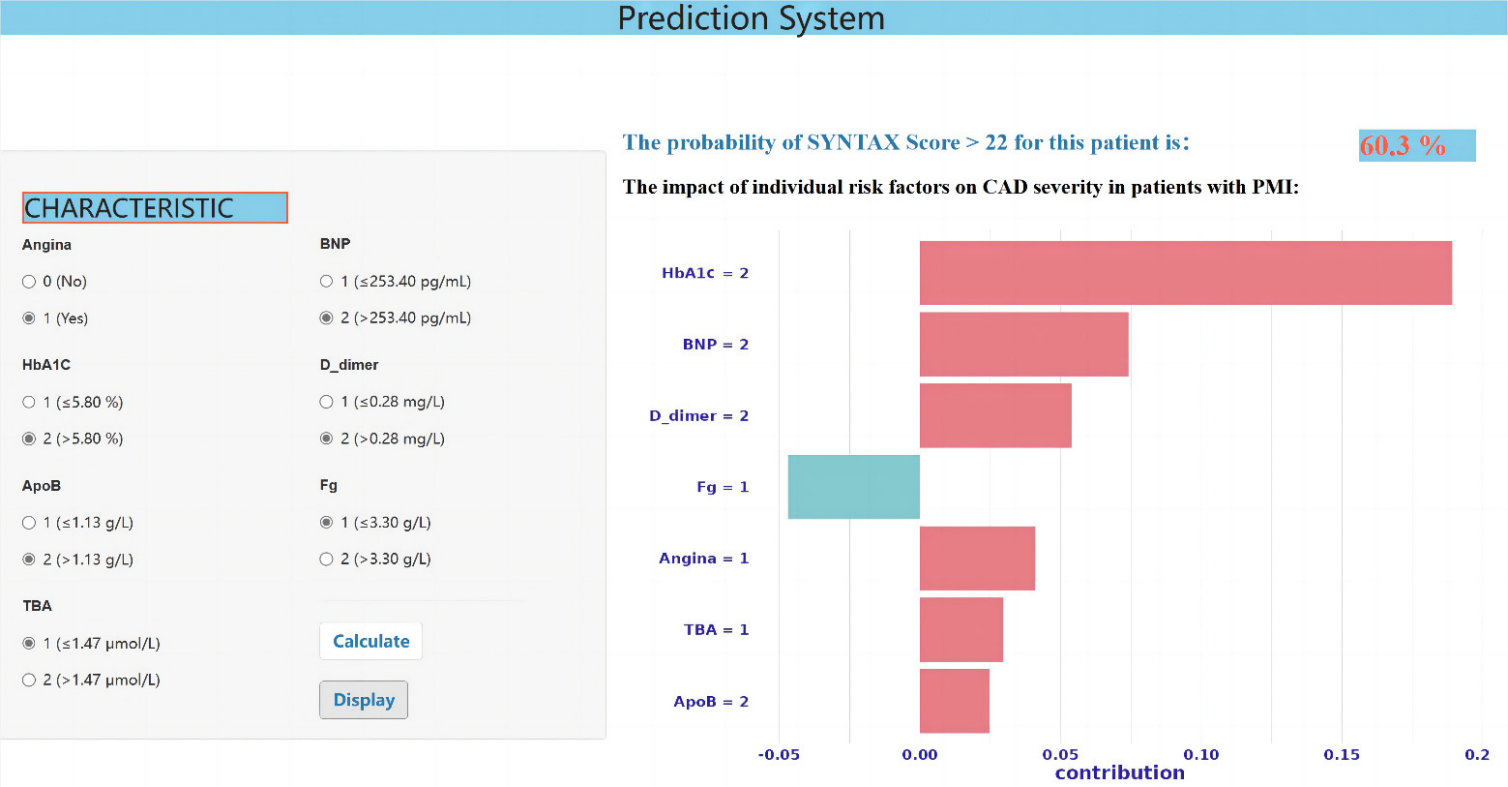
**The risk prediction system clinical interface indicates the probability of a SNYTAX score >22**. HbA1c, glycosylated hemoglobin; ApoB, apolipoprotein B; TBA, total bile acid; BNP, B-type natriuretic peptide; Fg, fibrinogen; CAD, coronary artery disease; PMI, premature myocardial infarction.

By establishing a prediction system for the severity of coronary artery lesions in patients with PMI, we can efficiently calculate the risk probability of patients having a SYNTAX score >22 based on their clinical laboratory indicators. Fig. 4 provides a vivid example of how, by selecting seven specific variables based on the clinical data of a patient with PMI, we can derive a 60.3% probability that the SYNTAX score exceeds 22. The online prediction system can be accessed through the following website: https://pmisyntax.shinyapps.io/appdecision/.

Moreover, individualized shapley additive explanation (SHAP) plots are utilized within the clinical interface of the prediction system to illustrate the selected variables visually. These plots illustrate the trend and extent to which the variable values influence and contribute to the overall results in the prediction system. SHAP is an algorithm employed for interpreting model predictions, where red represents variable values that negatively impact coronary artery lesions, while blue represents a relatively positive role.

## 4. Discussion

In recent years, there has been a significant increase in the availability of observational data for real-world studies (RWSs). As a result, machine learning techniques in the cardiovascular field have gained popularity, particularly in tasks such as image interpretation, risk identification, rational diagnosis, and prognosis prediction of cardiovascular diseases [23]. Compared to traditional statistical models, machine learning models have demonstrated superior predictive and discriminative abilities in certain studies [24, 25].

The five machine learning models in this study all have different strengths and weaknesses. Lasso–logistic optimizes the loss function of logistic regression with L1 regularization and selects the most important features to improve the performance of the model; however, it may not be able to capture the complex nonlinear relationship between the hypothesized features and the target variables in the model. RF is an integrated learning method with strong robustness; however, a single tree is not as explanatory as logistic regression for complex models and may require more computational resources and time for training and prediction. KNN performs classification or regression by calculating the distance between the test and training samples, which is suitable for small-scale datasets. However, KNN performs poorly when applied to large-scale datasets since it is more sensitive to noise and outliers. SVM can handle nonlinear classification problems by selecting appropriate kernel functions to map the data to higher dimensions. However, the kernel function and its parameter selection greatly impact the model performance, as may improper selection. XGBoost is a gradient-boosting method generated by constructing a series of decision trees; each iteration focuses on the error of the previous iteration. XGBoost improves the performance of the model by optimizing the objective function (including the loss function and the regularization term). The implementation enhances the accuracy and generalization of the final model by weighted learning and incremental improvement of the predictions. XGBoost can provide feature importance scores, which aid in understanding the model’s decisions. However, it requires tuning multiple hyperparameters (e.g., learning rate, tree depth, subsample ratio). In this study, with more categorical variables, the XGBoost model shows relatively good performance, depending on the fact that it is based on a decision tree model and employs several optimization strategies [26].

Compared with the nomogram prediction models established in existing studies [27, 28], our PMI patient prediction system is based on multiple machine learning algorithms and provides users with an interactive webpage, which can update the data in real-time and dynamically display the prediction results, providing intuitive trends. In addition, since subsequent studies will possess expanded sample sizes and increased variables, our prediction system supports the continuous updating and optimization of the model, as well as the integration of multiple data sources, thus improving the prediction accuracy comprehensively. In our team’s previous 6-year cohort follow-up study of patients with PMI, we found a 54% prevalence of metabolic syndrome (MS) in patients with PMI. Moreover, the percentage of multibranch lesions was higher in the metabolic syndrome group (62.7%). Furthermore, we observed that the prevalence of MACEs was 17.9%, and MS was an independent predictor of MACEs in PMI patients [29]. Therefore, we further explored the important factors affecting coronary artery pathology in PMI patients for primary and secondary prevention. The variables included in our prediction model, including HbA1c, angina, ApoB, TBA, BNP, D-dimer, and Fg, are readily available in clinical data and have been extensively studied for their adverse effects on cardiovascular disease.

A recent study evaluated the relationship between HbA1c and CAD severity using multifactorial logistic regression analysis, which showed that HbA1c was significantly associated with the presence and severity of CAD, consistent with our findings [30]. Moreover, a cross-sectional study revealed that patients with high HbA1c levels have a higher chance of developing coronary multibranch lesions [31]. In patients with dyslipidemia, those exhibiting elevated HbA1c levels were more prone to developing atherosclerotic dyslipidemia than those with lower HbA1c levels [32]. This finding provides insight for future research on coronary lesions and metabolism.

J C Kaski *et al*. [33] conducted a prospective study to investigate the significant impact of a history of angina in predicting the degree of coronary artery stenosis in acute coronary syndromes. They found that patients with a history of angina had a higher likelihood of experiencing vascular occlusion in the following months; this effect was particularly pronounced in patients with unstable angina.

Study have shown that D-dimer levels are higher in patients with severe lesions compared to those with milder coronary lesions in acute myocardial infarction. In fact, D-dimer has been identified as an independent predictor of coronary lesion severity in patients with myocardial infarction [34]. Additionally, a prospective study with a mean follow-up of up to 18 months found a correlation between plasma D-dimer levels and coronary lesion severity, even after adjusting for confounding factors [35].

In a study conducted on an African population with CAD, elevated levels of ApoB were observed with increasing severity of coronary lesions, and it was identified as an independent predictor of CAD severity [36]. A further comprehensive study has demonstrated that higher levels of ApoB are significantly associated with residual risk of coronary atherosclerotic heart disease and the severity of coronary atherosclerosis. Conversely, no such associations have been observed for elevated LDL-C levels [37].

In most current studies, many scholars have focused on the impact of BNP levels on the occurrence and prognosis of adverse cardiovascular disease outcomes, such as heart failure and cardiac structural remodeling [38, 39]. However, in a prospective, large-scale study involving the Han Chinese population, it was demonstrated that BNP levels were associated with the severity of coronary artery stenosis in CAD; furthermore, the BNP level was identified as a multivariate independent predictor of CAD in a logistic regression analysis [40]. Moreover, study have shown that a significant proportion of patients had multibranch vasculopathy when BNP levels exceeded 80 pg/mL [41].

Fg is involved in the development of vascular inflammation and atherosclerosis [42]. A recent study has demonstrated that elevated plasma Fg levels can serve as a predictor of severe coronary artery stenosis in young patients with myocardial infarction. Furthermore, multifactorial logistic regression analysis has shown that plasma Fg levels are an independent marker for predicting the presence and severity of coronary artery stenosis [43]. Moreover, Fg levels may be closely associated with the long-term prognosis of patients with myocardial infarction, providing a new avenue for long-term studies and risk stratification in patients with PMI [44].

Bile acids play a crucial role in lipid metabolism. Indeed, previous research has indicated that reduced bile acid production may result in the accumulation of cholesterol, thereby contributing to the progression of advanced atherosclerosis [45]. Recently, it was discovered that lower serum total bile acid levels are highly correlated with the severity of coronary artery lesions, myocardial injury, and inflammation, especially in patients with AMI. Lower serum bile acid levels may indicate more severe coronary artery lesions and a worse prognosis [46]. However, the results of Zhang *et al*. [47] suggested that coronary stenosis and high-risk coronary plaque severity augmented with increasing quartiles of serum total bile acid levels. The composition of total bile acid is complex, and theoretical research on this aspect is limited. Therefore, its mechanism in atherosclerosis remains incompletely understood, and further studies are necessary to explore the role of specific bile acids and the metabolomics of bile acids in the progression of cardiovascular disease.

The most important purpose of our prediction system is the preoperative evaluation of patients with PMI for coronary intervention. In patients with PMI in developed regions who are eligible for coronary intervention at presentation, when the prediction system suggests a high probability of the patient possessing a SYNTAX score >22, the surgeon needs to consider partial or complete revascularization in the context of the clinical situation because of multiple coronary branches or occlusive lesions. For patients with PMI in underdeveloped areas who are not eligible for coronary intervention, or for patients with PMI diagnosed at a later stage, when the prediction system suggests a high probability of a SYNTAX score >22, the first physician should intensify drug therapy, perform close electrocardiographic monitoring, and closely observe changes in the condition of the patient with PMI. The same applies even to PMI patients with onset in developed areas who refuse to undergo coronary intervention.

There are some limitations and prospects that should be considered in this study. First, our data were generated from an observational study conducted over 6 years at a single center, meaning the sample size needs to be increased further. Although we used cross-validation, we recognize the need to test the model on external datasets to confirm its applicability in various patient populations and settings. Our future research will focus on obtaining different datasets from multiple sources for more in-depth data training and testing, including other hospitals and geographic areas. Second, the clinical data of PMI patients in this study were collected from electronic medical records, and although consecutive enrollment of patients who met inclusion and exclusion criteria minimized selection bias, there may be a patient memory bias for medical history. Third, it was not possible to assess the potential impact of nontraditional risk factors such as lifestyle, psychological status, and genetic factors on coronary artery disease in patients with PMI. Fourth, because the study population included early-onset STEMI and NSTEMI, we did not include electrocardiographic data to predict the severity of coronary lesions in patients with PMI.

## 5. Conclusions

We developed a new and straightforward system for predicting the severity of lesions in patients with PMI. This system enables the identification of high-risk groups with severe lesions in PMI patients at an early stage, before coronary intervention. Furthermore, the system can also guide precise clinical management and decision-making.

## Data Availability

The datasets generated during and/or analyzed during the current study are available from the corresponding author on reasonable request.

## Abbreviations

AMI, acute myocardial infarction; PMI, premature myocardial infarction; RF, random forest; KNN, k-nearest neighbor; SVM, support vector machine; ROCs, receiver operating characteristic curves; AUC, area under the ROC curve; DCA, decision curve analysis; CAD, coronary artery disease; MI, myocardial infarction; WBCs, white blood cells; RBCs, red blood cells; Hb, hemoglobin; PLT, platelet; CRP, C-reactive protein; NLR, neutrophil-to-lymphocyte ratio; PLR, platelet-to-lymphocyte ratio; MLR, monocyte-to-lymphocyte ratio; CLR, C-reactive protein-to-lymphocyte ratio; SIRI, systemic inflammation response index; SII, systemic immune-inflammation index; HbA1c, glycosylated hemoglobin; Glu, glucose; TC, total cholesterol; TG, triglycerides; HDL, high-density lipoprotein; LDL, low-density lipoprotein; VLDL, very-low-density lipoprotein; non-HDL, non-high-density lipoprotein; RC, residual cholesterol; ApoA1, apolipoprotein A1; ApoB, apolipoprotein B; TyG, triglyceride glucose index; Cr, creatinine; UA, uric acid; TBA, total bile acid; TBil, total bilirubin; DBil, direct bilirubin; ALT, alanine aminotransferase; AST, aspartate aminotransferase; LDH, lactate dehydrogenase; α-HBDH, alpha-hydroxybutyrate dehydrogenase; HCY, homocysteine; CK, creatine kinase; CK-MB, creatine kinase MB; TnT, troponin T; BNP, B-type natriuretic peptide; Fg, fibrinogen.

## Supplementary Material

Supplementary material associated with this article can be found, in the online version, at https://doi.org/10.31083/RCM26102.
