# Supplementary material for: Advanced Machine Learning to Predict Coronary Artery Disease Severity in Patients with Premature Myocardial Infarction
Source: Rev Cardiovasc Med. 2025 Jan 16;26(1):26102. doi: 10.31083/RCM26102 (PMC11760553; doi:10.31083/RCM26102)
Supplement: Supplementary file 1 [file 2153-8174-26-1-26102-s1.docx]

Supplementary Table 1.The baseline table of raw continuous variables for laboratory data in PMI patients.

|  | **Overall (n = 1111)** | **SYNTAX ≤ 22  (n = 845)** | **SYNTAX ＞ 22  (n = 266)** | **p value** |
| --- | --- | --- | --- | --- |
| **Laboratory data** |  |  |  |  |
| **Blood routine** |  |  |  |  |
| WBC, 10^9/L | 10.2 (8.5,12.4) | 10.2 (8.5,12.4) | 10.3 (8.4,12.6) | 0.541 |
| Neutrophil, % | 73.4 (66.6,80.2) | 73.2 (66.6,80.1) | 74.2 (66.7,80.7) | 0.518 |
| Lymphocyte, % | 18.4 (13.1,24.8) | 18.7 (13.4,25.0) | 17.3 (12.8,24.6) | 0.211 |
| Monocyte, % | 6.0 (4.8,7.3) | 6.0 (4.8,7.3) | 6.2 (4.8,7.8) | 0.090 |
| Neutrophil, 10^9/L | 7.5 (5.7,9.7) | 7.5 (5.6,9.5) | 7.5 (5.8,10.1) | 0.540 |
| Lymphocyte, 10^9/L | 1.8 (1.4,2.3) | 1.8 (1.4,2.3) | 1.8 (1.4,2.3) | 0.414 |
| Monocyte, 10^9/L | 0.6 (0.5,0.8) | 0.6 (0.4,0.8) | 0.6 (0.5,0.8) | 0.074 |
| RBC, 10^12/L | 4.9 (4.5,5.2) | 4.9 (4.5,5.2) | 4.8 (4.5,5.2) | 0.475 |
| Hb, g/L | 148.0 (139.0,158.0) | 148.0 (139.0,158.0) | 146.0 (137.0,157.3) | 0.124 |
| PLT, 10^9/L | 240.0 (207.0,283.0) | 239.0 (206.0,282.5) | 244.0 (211.3,283.3) | 0.561 |
| **Inflammation indicators** |  |  |  |  |
| CRP, mg/L | 5.5 (2.4,12.5) | 5.2 (2.3,11.8) | 6.7 (2.8,14.9) | 0.016* |
| NLR | 4.0 (2.7,6.1) | 3.9 (2.7,6.0) | 4.2 (2.8,6.4) | 0.266 |
| PLR | 131.5 (99.5,176.0) | 131.3 (99.8,174.3) | 133.6 (98.8,181.4) | 0.407 |
| MLR | 0.3 (0.2,0.5) | 0.3 (0.2,0.4) | 0.4 (0.2,0.5) | 0.024* |
| CLR | 3.2 (1.3,7.7) | 3.1 (1.2,7.2) | 3.7 (1.6,9.8) | 0.008* |
| SIRI | 517.5 (273.5,1021.6) | 506.8 (268.7,994.5) | 558.4 (278.9,1045.8) | 0.294 |
| SII | 964.8 (619.8,1512.5) | 946.0 (606.8,1493.9) | 997.9 (644.1,1617.2) | 0.298 |
| **Glycolipid metabolism indicators** |  |  |  |  |
| HbA1C, % | 5.8 (5.6,6.3) | 5.8 (5.6,6.2) | 6.0 (5.8,6.8) | <0.001** |
| Glu, mmol/L | 5.8 (5.1,7.6) | 5.7 (5.0,7.2) | 6.0 (5.2,8.9) | 0.001* |
| TC, mmol/L | 4.8 (4.1,5.5) | 4.8 (4.1,5.4) | 4.9 (4.2,5.6) | 0.178 |
| TG, mmol/L | 2.0 (1.4,2.9) | 2.0 (1.4,3.0) | 2.0 (1.5,2.7) | 0.738 |
| HDLC, mmol/L | 0.9 (0.8,1.1) | 0.9 (0.8,1.1) | 0.9 (0.8,1.1) | 0.655 |
| LDLC, mmol/L | 3.2 (2.5,3.8) | 3.2 (2.5,3.8) | 3.3 (2.6,3.9) | 0.062 |
| VLDLC, mmol/L | 0.6 (0.4,0.8) | 0.6 (0.4,0.8) | 0.6 (0.4,0.8) | 0.483 |
| non-HDLC, mmol/L | 3.9 (3.2,4.5) | 3.8 (3.1,4.5) | 3.9 (3.2,4.6) | 0.142 |
| RC, mmol/L | 0.6 (0.4,0.8) | 0.6 (0.4,0.8) | 0.6 (0.4,0.8) | 0.483 |
| TC/HDLC | 5.2 (4.3,6.3) | 5.2 (4.2,6.3) | 5.3 (4.3,6.2) | 0.510 |
| TG/HDLC | 2.2 (1.4,3.4) | 2.2 (1.4,3.5) | 2.2 (1.5,3.2) | 0.601 |
| LDLC/HDLC | 3.5 (2.7,4.3) | 3.4 (2.7,4.2) | 3.5 (2.8,4.4) | 0.198 |
| Apo A1, g/L | 1.1 (1.0,1.2) | 1.1 (1.0,1.2) | 1.1 (1.0,1.2) | 0.758 |
| Apo B, g/L | 1.1 (0.9,1.3) | 1.1 (0.9,1.3) | 1.2 (1.0,1.4) | 0.003* |
| Apo A1/Apo B | 1.0 (0.8,1.2) | 1.0 (0.8,1.2) | 1.0 (0.8,1.1) | 0.012* |
| TyG | 9.2 (8.8,9.7) | 9.2 (8.8,9.7) | 9.2 (8.8,9.8) | 0.123 |
| **Kidney function indicators** |  |  |  |  |
| Urea, mmol/L | 4.3 (3.5,5.4) | 4.3 (3.5,5.3) | 4.4 (3.6,5.4) | 0.076 |
| Cr, umol/L | 75.0 (66.0,85.0) | 75.0 (66.0,85.0) | 76.0 (65.8,87.0) | 0.365 |
| UA, umol/L | 360.0 (295.0,429.0) | 361.0 (294.5,429.0) | 355.0 (297.8,429.0) | 0.710 |
| **Liver function indicators** |  |  |  |  |
| TBA, umol/L | 1.5 (0.9,2.4) | 1.5 (0.9,2.4) | 1.3 (0.8,2.3) | 0.034* |
| TBil, umol/L | 13.8 (9.9,18.9) | 14.0 (10.0,18.8) | 13.4 (9.9,19.4) | 0.981 |
| DBil, umol/L | 5.0 (3.9,6.5) | 5.0 (3.9,6.5) | 5.0 (4.0,6.7) | 0.879 |
| ALT, U/L | 42.8 (28.3,68.0) | 42.4 (28.3,67.5) | 44.1 (28.8,69.1) | 0.723 |
| AST, U/L | 106.4 (45.6,220.9) | 106.2 (47.3,214.2) | 115.6 (41.6,246.1) | 0.561 |
| LDH, U/L | 439.0 (261.0,782.0) | 429.0 (258.5,740.0) | 480.0 (275.8,897.3) | 0.049* |
| α-HBDH, U/L | 401.0 (217.0,782.0) | 392.5 (214.5,705.5) | 435.0 (236.0,885.5) | 0.046* |
| **HCY, umol/L** | 12.7 (10.1,18.2) | 12.7 (10.0,18.9) | 13.0 (10.3,17.3) | 0.735 |
| **Cardiac Function Indicators** |  |  |  |  |
| CK, U/L | 1016.0 (331.0,2158.0) | 1005.0 (344.0,2038.5) | 1064.0 (287.3,2794.5) | 0.393 |
| CK-MB, U/L | 88.0 (34.0,184.0) | 88.0 (35.0,173.5) | 88.0 (29.8,205.5) | 0.735 |
| TnT, ug/L | 1.9 (0.6,4.5) | 1.9 (0.6,4.3) | 2.0 (0.6,5.4) | 0.224 |
| BNP, pg/mL | 253.4 (101.9,644.7) | 246.6 (87.7,564.4) | 362.3 (162.6,855.5) | <0.001** |
| **Coagulation indicators** |  |  |  |  |
| D-dimer, mg/L | 0.3 (0.2,0.4) | 0.3 (0.2,0.4) | 0.3 (0.2,0.5) | 0.016* |
| Fg, g/L | 3.3 (2.9,3.9) | 3.3 (2.8,3.8) | 3.5 (3.0,4.1) | <0.001** |
| Notes: WBC, white blood cells; RBC, red blood cells; Hb, hemoglobin; PLT, platelet; CRP, C-reactive protein; NLR, neutrophil to lymphocyte ratio; PLR, platelet to lymphocyte ratio; MLR, monocyte to lymphocyte ratio; CLR, C-reactive protein to lymphocyte ratio; SIRI, systemic inflammation response index; SII, systemic immune-inflammation index; HbA1C, glycosylated hemoglobin; Glu, glucose; TC, total cholesterol; TG, triglycerides; HDL, high-density lipoprotein; LDL, low-density lipoprotein; VLDL, very-low-density lipoprotein; non-HDL, non-high-density lipoprotein; RC, residual cholesterol; Apo A1, apolipoprotein A1; Apo B, apolipoprotein B; TyG, triglyceride glucose index; Cr, creatinine; UA, uric acid; TBA, total bile acid; TBil, total bilirubin; DBil, direct bilirubin; ALT, alanine aminotransferase; AST, aspartate aminotransferase; LDH, lactate dehydrogenase; α-HBDH, alpha-hydroxybutyrate dehydrogenase; HCY, homocysteine; CK, creatine kinase; CK-MB, creatine kinase MB; TnT, troponin T; BNP, B-type natriuretic peptide; Fg, fibrinogen.(*p < 0.05,**p < 0.001) | | | | |

Supplementary Table 2.Baseline characteristics for both the training and test sets of the PMI patients according to SYNTAX score.

| **Variables** | **Training set** | | | **Test set** | | |
| --- | --- | --- | --- | --- | --- | --- |
|  | **SYNTAX≤22 (n=591)** | **SYNTAX＞22 (n=186)** | **p value** | **SYNTAX≤22 (n=254)** | **SYNTAX＞22 (n=80)** | **p value** |
| Age＞43years, (%) | 248 (42.0) | 85 (45.7) | 0.416 | 116 (45.7) | 41 (51.2) | 0.457 |
| Male, (%) | 542 (91.7) | 168 (90.3) | 0.662 | 233 (91.7) | 70 (87.5) | 0.359 |
| Smoking, (%) | 419 (70.9) | 126 (67.7) | 0.467 | 181 (71.3) | 59 (73.8) | 0.772 |
| Drinking, (%) | 227 (38.4) | 54 (29.0) | 0.025* | 100 (39.4) | 28 (35.0) | 0.569 |
| **Past history, (%)** |  |  |  |  |  |  |
| Hypertension | 286 (48.4) | 90 (48.4) | 1.000 | 120 (47.2) | 37 (46.2) | 0.979 |
| Diabetes | 104 (17.6) | 50 (26.9) | 0.008* | 59 (23.2) | 22 (27.5) | 0.530 |
| Hyperlipidemia | 152 (25.7) | 48 (25.8) | 1.000 | 51 (20.1) | 25 (31.2) | 0.054 |
| Stroke | 21 (3.6) | 7 (3.8) | 1.000 | 5 (2.0) | 5 (6.2) | 0.113 |
| Angina | 84 (14.2) | 41 (22.0) | 0.016* | 32 (12.6) | 21 (26.2) | 0.006* |
| Family history of CAD | 68 (11.5) | 18 (9.7) | 0.576 | 23 (9.1) | 8 (10.0) | 0.974 |
| **Killip classification, (%)** |  |  | 0.418 |  |  | 0.534 |
| I | 572 (96.8) | 177 (95.2) |  | 247 (97.2) | 76 (95.0) |  |
| ≥II | 19 (3.2) | 9 (4.8) |  | 7 (2.8) | 4 (5.0) |  |
| **Type of MI, (%)** |  |  | 1.000 |  |  | 0.737 |
| STEMI | 466 (78.8) | 147 (79.0) |  | 191 (75.2) | 58 (72.5) |  |
| NSTEMI | 125 (21.2) | 39 (21.0) |  | 63 (24.8) | 22 (27.5) |  |
| **Laboratory data** |  |  |  |  |  |  |
| **Blood routine** |  |  |  |  |  |  |
| WBC＞10.23*10^9/L, (%) | 294 (49.7) | 92 (49.5) | 1.000 | 127 (50.0) | 42 (52.5) | 0.793 |
| Neutrophil%＞73.43%, (%) | 297 (50.3) | 89 (47.8) | 0.626 | 121 (47.6) | 48 (60.0) | 0.072 |
| Lymphocyte%＞18.40%, (%) | 300 (50.8) | 90 (48.4) | 0.631 | 131 (51.6) | 33 (41.2) | 0.138 |
| Monocyte%＞6.00%, (%) | 282 (47.7) | 101 (54.3) | 0.138 | 125 (49.2) | 43 (53.8) | 0.562 |
| Neutrophil＞7.50*10^9/L, (%) | 295 (49.9) | 93 (50.0) | 1.000 | 124 (48.8) | 42 (52.5) | 0.656 |
| Lymphocyte＞1.83*10^9/L, (%) | 299 (50.6) | 86 (46.2) | 0.341 | 131 (51.6) | 40 (50.0) | 0.906 |
| Monocyte＞0.60*10^9/L, (%) | 266 (45.0) | 95 (51.1) | 0.173 | 114 (44.9) | 41 (51.2) | 0.386 |
| RBC＞4.88*10^12/L, (%) | 289 (48.9) | 92 (49.5) | 0.960 | 137 (53.9) | 31 (38.8) | 0.025* |
| Hb＞148.00g/L, (%) | 284 (48.1) | 86 (46.2) | 0.727 | 134 (52.8) | 29 (36.2) | 0.014* |
| PLT＞240.00*10^9/L, (%) | 281 (47.5) | 101 (54.3) | 0.128 | 136 (53.5) | 36 (45.0) | 0.228 |
| **Inflammation indicators** |  |  |  |  |  |  |
| CRP＞5.47mg/L, (%) | 283 (47.9) | 102 (54.8) | 0.116 | 115 (45.3) | 53 (66.2) | 0.002* |
| NLR＞4.00, (%) | 290 (49.1) | 95 (51.1) | 0.694 | 122 (48.0) | 47 (58.8) | 0.123 |
| PLR＞131.49, (%) | 296 (50.1) | 92 (49.5) | 0.949 | 126 (49.6) | 42 (52.5) | 0.747 |
| MLR＞0.33, (%) | 283 (47.9) | 102 (54.8) | 0.116 | 126 (49.6) | 44 (55.0) | 0.476 |
| CLR＞3.11, (%) | 293 (49.6) | 99 (53.2) | 0.433 | 114 (44.9) | 49 (61.3) | 0.015* |
| SIRI＞2.48, (%) | 289 (48.9) | 96 (51.6) | 0.575 | 127 (50.0) | 44 (55.0) | 0.514 |
| SII＞960.73, (%) | 282 (47.7) | 93 (50.0) | 0.646 | 136 (53.5) | 45 (56.2) | 0.768 |
| **Glycolipid metabolism indicators** |  |  |  |  |  |  |
| HbA1C＞5.80%, (%) | 172 (29.1) | 132 (71.0) | <0.001** | 98 (38.6) | 64 (80.0) | <0.001** |
| Glu＞5.77mmol/L, (%) | 272 (46.0) | 105 (56.5) | 0.017* | 135 (53.1) | 44 (55.0) | 0.872 |
| TC＞4.81mmol/L, (%) | 295 (49.9) | 105 (56.5) | 0.141 | 118 (46.5) | 34 (42.5) | 0.623 |
| TG＞2.02mmol/L, (%) | 297 (50.3) | 90 (48.4) | 0.719 | 123 (48.4) | 38 (47.5) | 0.987 |
| HDLC＞0.92mmol/L, (%) | 293 (49.6) | 98 (52.7) | 0.512 | 107 (42.1) | 38 (47.5) | 0.474 |
| LDLC＞3.19mmol/L, (%) | 288 (48.7) | 109 (58.6) | 0.024* | 120 (47.2) | 38 (47.5) | 1.000 |
| VLDLC＞0.56mmol/L, (%) | 289 (48.9) | 90 (48.4) | 0.970 | 121 (47.6) | 35 (43.8) | 0.632 |
| non-HDLC＞3.86mmol/L, (%) | 288 (48.7) | 100 (53.8) | 0.266 | 124 (48.8) | 42 (52.5) | 0.656 |
| RC＞0.56mmol/L, (%) | 293 (49.6) | 91 (48.9) | 0.943 | 123 (48.4) | 35 (43.8) | 0.547 |
| TC/HDLC＞5.21, (%) | 295 (49.9) | 89 (47.8) | 0.684 | 132 (52.0) | 40 (50.0) | 0.858 |
| TG/HDLC＞2.21, (%) | 284 (48.1) | 99 (53.2) | 0.252 | 133 (52.4) | 40 (50.0) | 0.810 |
| LDLC/HDLC＞3.46, (%) | 290 (49.1) | 102 (54.8) | 0.198 | 127 (50.0) | 38 (47.5) | 0.793 |
| Apo A1＞1.10g/L, (%) | 269 (45.5) | 87 (46.8) | 0.829 | 121 (47.6) | 35 (43.8) | 0.632 |
| Apo B＞1.13g/L, (%) | 273 (46.2) | 114 (61.3) | <0.001** | 113 (44.5) | 45 (56.2) | 0.087 |
| Apo A1/Apo B＞0.98, (%) | 321 (54.3) | 83 (44.6) | 0.026* | 139 (54.7) | 37 (46.2) | 0.232 |
| TyG＞9.18, (%) | 286 (48.4) | 99 (53.2) | 0.287 | 128 (50.4) | 42 (52.5) | 0.841 |
| **Kidney function indicators** |  |  |  |  |  |  |
| Urea＞4.30mmol/L, (%) | 280 (47.4) | 104 (55.9) | 0.052 | 122 (48.0) | 36 (45.0) | 0.730 |
| Cr＞75.00umol/L, (%) | 280 (47.4) | 105 (56.5) | 0.038* | 131 (51.6) | 38 (47.5) | 0.612 |
| UA＞360.00umol/L, (%) | 301 (50.9) | 88 (47.3) | 0.437 | 131 (51.6) | 34 (42.5) | 0.198 |
| **Liver function indicators** |  |  |  |  |  |  |
| TBA＞1.47umol/L, (%) | 313 (53.0) | 83 (44.6) | 0.057 | 129 (50.8) | 28 (35.0) | 0.019* |
| TBil＞13.80umol/L, (%) | 299 (50.6) | 88 (47.3) | 0.486 | 130 (51.2) | 35 (43.8) | 0.302 |
| DBil＞5.00umol/L, (%) | 271 (45.9) | 83 (44.6) | 0.834 | 110 (43.3) | 32 (40.0) | 0.695 |
| ALT＞42.80U/L, (%) | 291 (49.2) | 97 (52.2) | 0.543 | 124 (48.8) | 42 (52.5) | 0.656 |
| AST＞106.55U/L, (%) | 306 (51.8) | 100 (53.8) | 0.697 | 113 (44.5) | 36 (45.0) | 1.000 |
| LDH＞438.00U/L, (%) | 289 (48.9) | 103 (55.4) | 0.145 | 122 (48.0) | 42 (52.5) | 0.569 |
| α-HBDH＞400.50U/L, (%) | 293 (49.6) | 103 (55.4) | 0.195 | 120 (47.2) | 40 (50.0) | 0.763 |
| **HCY＞12.70umol/L, (%)** | 271 (45.9) | 104 (55.9) | 0.021* | 132 (52.0) | 41 (51.2) | 1.000 |
| **Cardiac Function Indicators** |  |  |  |  |  |  |
| CK＞1017U/L, (%) | 300 (50.8) | 98 (52.7) | 0.708 | 118 (46.5) | 39 (48.8) | 0.818 |
| CK-MB＞88U/L, (%) | 296 (50.1) | 93 (50.0) | 1.000 | 125 (49.2) | 37 (46.2) | 0.738 |
| TnT＞1.94ug/L, (%) | 299 (50.6) | 99 (53.2) | 0.587 | 117 (46.1) | 39 (48.8) | 0.771 |
| BNP＞269.75pg/mL, (%) | 267 (45.2) | 115 (61.8) | <0.001** | 118 (46.5) | 54 (67.5) | 0.002* |
| **Coagulation indicators** |  |  |  |  |  |  |
| D-dimer＞0.28mg/L, (%) | 251 (42.5) | 118 (63.4) | <0.001** | 119 (46.9) | 46 (57.5) | 0.125 |
| Fg＞3.30g/L, (%) | 267 (45.2) | 118 (63.4) | <0.001** | 119 (46.9) | 52 (65.0) | 0.007* |
| Notes: CAD, coronary artery disease; MI, myocardial infarction; WBC, white blood cells; RBC, red blood cells; Hb, hemoglobin; PLT, platelet; CRP, C-reactive protein; NLR, neutrophil to lymphocyte ratio; PLR, platelet to lymphocyte ratio; MLR, monocyte to lymphocyte ratio; CLR, C-reactive protein to lymphocyte ratio; SIRI, systemic inflammation response index; SII, systemic immune-inflammation index; HbA1C, glycosylated hemoglobin; Glu, glucose; TC, total cholesterol; TG, triglycerides; HDL, high-density lipoprotein; LDL, low-density lipoprotein; VLDL, very-low-density lipoprotein; non-HDL, non-high-density lipoprotein; RC, residual cholesterol; Apo A1, apolipoprotein A1; Apo B, apolipoprotein B; TyG, triglyceride glucose index; Cr, creatinine; UA, uric acid; TBA, total bile acid; TBil, total bilirubin; DBil, direct bilirubin; ALT, alanine aminotransferase; AST, aspartate aminotransferase; LDH, lactate dehydrogenase; α-HBDH, alpha-hydroxybutyrate dehydrogenase; HCY, homocysteine; CK, creatine kinase; CK-MB, creatine kinase MB; TnT, troponin T; BNP, B-type natriuretic peptide; Fg, fibrinogen.(*p < 0.05,**p < 0.001) | | | | | | |

Supplementary Table 3.Comparison of baseline characteristics between training set and test set.

| **Variables** | **Training set (n=777)** | **Test set (n=334)** | **p value** |
| --- | --- | --- | --- |
| **SYNTAX score >22, (%)** | 186 (23.9) | 80 (24.0) | 1.000 |
| Age＞43years, (%) | 333 (42.9) | 157 (47.0) | 0.226 |
| Male, (%) | 710 (91.4) | 303 (90.7) | 0.811 |
| Smoking, (%) | 545 (70.1) | 240 (71.9) | 0.614 |
| Drinking, (%) | 281 (36.2) | 128 (38.3) | 0.538 |
| **Past history, (%)** |  |  |  |
| Hypertension | 376 (48.4) | 157 (47.0) | 0.720 |
| Diabetes | 154 (19.8) | 81 (24.3) | 0.114 |
| Hyperlipidemia | 200 (25.7) | 76 (22.8) | 0.327 |
| Stroke | 28 (3.6) | 10 (3.0) | 0.739 |
| Angina | 125 (16.1) | 53 (15.9) | 0.998 |
| Family history of CAD | 86 (11.1) | 31 (9.3) | 0.434 |
| **Killip classification, (%)** |  |  | 0.936 |
| I | 749 (96.4) | 323 (96.7) |  |
| ≥II | 28 (3.6) | 11 (3.3) |  |
| **Type of MI, (%)** |  |  | 0.130 |
| STEMI | 613 (78.9) | 249 (74.6) |  |
| NSTEMI | 164 (21.1) | 85 (25.4) |  |
| **Laboratory data** |  |  |  |
| **Blood routine** |  |  |  |
| WBC＞10.23*10^9/L, (%) | 386 (49.7) | 169 (50.6) | 0.829 |
| Neutrophil%＞73.43%, (%) | 386 (49.7) | 169 (50.6) | 0.829 |
| Lymphocyte%＞18.40%, (%) | 390 (50.2) | 164 (49.1) | 0.789 |
| Monocyte%＞6.00%, (%) | 383 (49.3) | 168 (50.3) | 0.808 |
| Neutrophil＞7.50*10^9/L, (%) | 388 (49.9) | 166 (49.7) | 0.995 |
| Lymphocyte＞1.83*10^9/L, (%) | 385 (49.5) | 171 (51.2) | 0.661 |
| Monocyte＞0.60*10^9/L, (%) | 361 (46.5) | 155 (46.4) | 1.000 |
| RBC＞4.88*10^12/L, (%) | 381 (49.0) | 168 (50.3) | 0.748 |
| Hb＞148.00g/L, (%) | 370 (47.6) | 163 (48.8) | 0.767 |
| PLT＞240.00*10^9/L, (%) | 382 (49.2) | 172 (51.5) | 0.517 |
| **Inflammation indicators** |  |  |  |
| CRP＞5.47mg/L, (%) | 385 (49.5) | 168 (50.3) | 0.870 |
| NLR＞4.00, (%) | 385 (49.5) | 169 (50.6) | 0.798 |
| PLR＞131.49, (%) | 388 (49.9) | 168 (50.3) | 0.964 |
| MLR＞0.33, (%) | 385 (49.5) | 170 (50.9) | 0.729 |
| CLR＞3.11, (%) | 392 (50.5) | 163 (48.8) | 0.661 |
| SIRI＞2.48, (%) | 385 (49.5) | 171 (51.2) | 0.661 |
| SII＞960.73, (%) | 375 (48.3) | 181 (54.2) | 0.081 |
| **Glycolipid metabolism indicators** |  |  |  |
| HbA1C＞5.80%, (%) | 304 (39.1) | 162 (48.5) | 0.005* |
| Glu＞5.77mmol/L, (%) | 377 (48.5) | 179 (53.6) | 0.137 |
| TC＞4.81mmol/L, (%) | 400 (51.5) | 152 (45.5) | 0.078 |
| TG＞2.02mmol/L, (%) | 387 (49.8) | 161 (48.2) | 0.671 |
| HDLC＞0.92mmol/L, (%) | 391 (50.3) | 145 (43.4) | 0.041* |
| LDLC＞3.19mmol/L, (%) | 397 (51.1) | 158 (47.3) | 0.275 |
| VLDLC＞0.56mmol/L, (%) | 379 (48.8) | 156 (46.7) | 0.570 |
| non-HDLC＞3.86mmol/L, (%) | 388 (49.9) | 166 (49.7) | 0.995 |
| RC＞0.56mmol/L, (%) | 384 (49.4) | 158 (47.3) | 0.561 |
| TC/HDLC＞5.21, (%) | 384 (49.4) | 172 (51.5) | 0.569 |
| TG/HDLC＞2.21, (%) | 383 (49.3) | 173 (51.8) | 0.484 |
| LDLC/HDLC＞3.46, (%) | 392 (50.5) | 165 (49.4) | 0.798 |
| Apo A1＞1.10g/L, (%) | 356 (45.8) | 156 (46.7) | 0.836 |
| Apo B＞1.13g/L, (%) | 387 (49.8) | 158 (47.3) | 0.484 |
| Apo A1/Apo B＞0.98, (%) | 404 (52.0) | 176 (52.7) | 0.882 |
| TyG＞9.18, (%) | 385 (49.5) | 170 (50.9) | 0.729 |
| **Kidney function indicators** |  |  |  |
| Urea＞4.30mmol/L, (%) | 384 (49.4) | 158 (47.3) | 0.561 |
| Cr＞75.00umol/L, (%) | 385 (49.5) | 169 (50.6) | 0.798 |
| UA＞360.00umol/L, (%) | 389 (50.1) | 165 (49.4) | 0.891 |
| **Liver function indicators** |  |  |  |
| TBA＞1.47umol/L, (%) | 396 (51.0) | 157 (47.0) | 0.252 |
| TBil＞13.80umol/L, (%) | 387 (49.8) | 165 (49.4) | 0.953 |
| DBil＞5.00umol/L, (%) | 354 (45.6) | 142 (42.5) | 0.384 |
| ALT＞42.80U/L, (%) | 388 (49.9) | 166 (49.7) | 0.995 |
| AST＞106.55U/L, (%) | 406 (52.3) | 149 (44.6) | 0.023* |
| LDH＞438.00U/L, (%) | 392 (50.5) | 164 (49.1) | 0.729 |
| α-HBDH＞400.50U/L, (%) | 396 (51.0) | 160 (47.9) | 0.384 |
| **HCY＞12.70umol/L, (%)** | 375 (48.3) | 173 (51.8) | 0.310 |
| **Cardiac Function Indicators** |  |  |  |
| CK＞1017U/L, (%) | 398 (51.2) | 157 (47.0) | 0.221 |
| CK-MB＞88U/L, (%) | 389 (50.1) | 162 (48.5) | 0.680 |
| TnT＞1.94ug/L, (%) | 398 (51.2) | 156 (46.7) | 0.189 |
| BNP＞269.75pg/mL, (%) | 382 (49.2) | 172 (51.5) | 0.517 |
| **Coagulation indicators** |  |  |  |
| D-dimer＞0.28mg/L, (%) | 369 (47.5) | 165 (49.4) | 0.604 |
| Fg＞3.30g/L, (%) | 385 (49.5) | 171 (51.2) | 0.661 |
| Notes: CAD, coronary artery disease; MI, myocardial infarction; WBC, white blood cells; RBC, red blood cells; Hb, hemoglobin; PLT, platelet; CRP, C-reactive protein; NLR, neutrophil to lymphocyte ratio; PLR, platelet to lymphocyte ratio; MLR, monocyte to lymphocyte ratio; CLR, C-reactive protein to lymphocyte ratio; SIRI, systemic inflammation response index; SII, systemic immune-inflammation index; HbA1C, glycosylated hemoglobin; Glu, glucose; TC, total cholesterol; TG, triglycerides; HDL, high-density lipoprotein; LDL, low-density lipoprotein; VLDL, very-low-density lipoprotein; non-HDL, non-high-density lipoprotein; RC, residual cholesterol; Apo A1, apolipoprotein A1; Apo B, apolipoprotein B; TyG, triglyceride glucose index; Cr, creatinine; UA, uric acid; TBA, total bile acid; TBil, total bilirubin; DBil, direct bilirubin; ALT, alanine aminotransferase; AST, aspartate aminotransferase; LDH, lactate dehydrogenase; α-HBDH, alpha-hydroxybutyrate dehydrogenase; HCY, homocysteine; CK, creatine kinase; CK-MB, creatine kinase MB; TnT, troponin T; BNP, B-type natriuretic peptide; Fg, fibrinogen.(*p < 0.05,**p < 0.001) | | | |
